# Patient-related factors that link chronic kidney disease and periodontitis: a scoping review

**DOI:** 10.1007/s10266-024-01031-y

**Published:** 2024-12-09

**Authors:** Kübra Kaymaz, Lluís Brunet-Llobet, María Dolores Rocha-Eiroa, Albert Ramírez-Rámiz, Muhiddin Abdi Mahmoud, Elias Isaack Mashala, Jaume Miranda-Rius

**Affiliations:** 1 Master of Public Health, School of Public Health, Faculty of Medicine, Private Dental Practice, Aberdeen, UK; 2 Department of Odontostomatology, Faculty of Medicine and Health Sciences, University of Barcelona, Barcelona, Spain; 3 Department of Dentistry, Hospital Sant Joan de Déu, University of Barcelona, Barcelona, Spain; 4 Hospital Dentistry and Periodontal Medicine Research Group. Institut de Recerca Sant Joan de Déu (IRSJD), Barcelona, Spain; 5 Department of Nephrology and Renal Transplantation, Mnazi Mmoja Referral Hospital, Zanzibar, Tanzania; 6 Doctoral Programme in Medicine and Translational Research, Faculty of Medicine and Health Sciences, Hospital Sant Joan de Déu, University of Barcelona, Barcelona, Spain

**Keywords:** Subgingival disbiotic biofilm, Periodontitis, Chronic kidney disease, Chronic inflammatory diseases, Kidney failure, Bleeding periodontal pockets, Systemic inflammation

## Abstract

Several studies have proposed the existence of an association between periodontitis and chronic kidney disease (CKD) based on biological premises. There is growing evidence that chronic inflammation caused by periodontitis may contribute to the progression of CKD. The present study aimed to investigate studies that link CKD and periodontitis, including periodontitis proxies such as oral hygiene and tooth loss, and patient-related factors such as inflammatory response and genetic polymorphisms. An electronic search was conducted on the MEDLINE (Pubmed), Cochrane Central Register of Controlled Trials (CENTRAL), Scopus, and Web of Science databases using an advanced search option up until August 2024. Thirty-two studies were included: 4 interventional, 16 cohort, and 12 case–control. Overall, the prevalence of periodontitis was significantly higher in patients with CKD: the diagnosis of periodontal disease was associated with an increase in the risk of incident CKD, and parameters of periodontal disease were negatively correlated with kidney function. Inside the field of periodontal medicine, the current evidence indicates a possible association between CKD and periodontitis and supports future longitudinal studies to investigate the two-way relationship between the diseases and their pathophysiology, and possibly to establish cause and effect.

## Introduction

Chronic kidney disease (CKD) is one of the most prevalent chronic illnesses worldwide. CKD affects 8% to 16% of the world’s population and claimed 1.2 million lives globally in 2017 [1, 2]. Most people who are affected are asymptomatic and may never be aware that they have the disease [3, 4]. Damage to the kidney brought on by a number of illnesses, particularly diabetes mellitus, hypertension, and glomerulonephritis, may evolve into CKD [2]. In 2017, CKD caused 3.58 million disability-adjusted life years (DALYs), over a third due to diabetic nephropathy [5]. CKD is indicated by a glomerular filtration rate (GFR) of less than 60 mL/min/1.73 m^2^, significant proteinuria (e.g., albuminuria of at least 30 mg per 24 h), or signs of kidney damage such as haematuria or structural abnormalities like polycystic or dysplastic kidney persisting for more than 3 months [6]. Other risk factors such as genetic issues should also be considered due to their influence on the appearance of CKD [7]. Also, chronic inflammation has been linked to the emergence of CKD in otherwise healthy individuals [7]. CKD is a multisystem disease process that has widespread effects on immune system activity, endothelial cell function, cognitive function, appetite, and emotional state [8, 9]. Anaemia, acidosis, blood electrolyte disturbances, abnormal bone metabolism, and the inability to clear uremic toxins are some of its hallmark signs [10–12].

The two main causes of mortality in people with CKD are cardiovascular diseases and infections, both of which are relatively common and are responsible for up to 70% of all-cause mortality among CKD patients [13, 14]. Around the world, more than one in every 1000 people has stage 5 or end-stage kidney disease, and 3 million require kidney transplant, peritoneal dialysis, or haemodialysis as part of their renal replacement therapy [15, 16], a treatment that entails significant healthcare costs. CKD is becoming more common, particularly in developing countries where up to now access to renal healthcare has been limited [17, 18].

Periodontitis is characterised by inflammation of the teeth-surrounding tissues that is host-mediated and is linked with oral microorganisms. With an overall global prevalence of up to 50%, and a prevalence of severe forms of roughly 11%, periodontal diseases are a major public health concern [19]. In 2018, the direct and indirect costs of periodontal disease were estimated to be $154.06 billion in the United States and €158.64 billion in Europe [20]. Patients with periodontal disorders are more likely to have tooth loss, edentulism, and masticatory dysfunction, all of which have a detrimental effect on their ability to eat well, live a fulfilling life, and feel confident in themselves [21]. Activation of host-derived proteinases is the end result of the molecular pathways of its pathogenesis, which causes the junctional epithelium to migrate apically and the bacterial biofilm to extend along the root surface, and also leads to the loss of marginal periodontal ligament fibres [22]. However, the onset and development of periodontitis depend on dysbiotic ecological changes in the microbiome in response to nutrients from gingival inflammatory and tissue breakdown products [23]. Thus, periodontitis is an oral dysbiosis-associated immune-mediated inflammatory disease [22, 24, 25]. The most recent research supports a multifactorial risk hypothesis, for instance smoking and diabetes, with numerous immunoinflammatory responses that increase the likelihood of dysbiotic microbiome alterations in genetically susceptible individuals, accelerating the initiation of the disease and increasing its severity.

Periodontitis is a chronic non-communicable disease (NCD) that shares risk factors with other NCDs, such as type II diabetes mellitus, and has a close relationship with cardiovascular and overall health [26]. Globally, there is growing recognition that better periodontal health will have positive effects on systemic well-being and health in general [27, 28]. Numerous investigations have revealed a link between periodontitis and systemic diseases such as type I and type II diabetes mellitus, cardiovascular disease, and CKD [29].

With regard to the patient-related factors in periodontitis and systemic diseases and to the two-way relationship between them, the primary mechanisms that cause systemic inflammation include the effects of periodontal bacteria (whether directly or indirectly). From diseased or inflamed periodontal tissues to other body regions, there is a systemic increase in inflammatory mediators due to periodontal inflammation and/or patient-related immune factors [30]. Interestingly, age, smoking, and poorly managed diabetes mellitus are associated with both periodontitis and with CKD [31–33]. Several studies have proposed an association between periodontitis and CKD based on biological assumptions [34]. There is also growing evidence that the chronic inflammation caused by periodontitis and the dysbiotic microbiome may lead to the progression of CKD [35].

Against this background, the present review systematically investigates studies that link CKD and periodontitis, assessing periodontitis proxies such as oral hygiene and tooth loss, and host-related factors including inflammatory response and genetic polymorphisms.

## Materials and methods

### Search strategy

An E-search up until August 2024 was conducted using an advanced search: MEDLINE (PubMed), Cochrane Central Register of Controlled Trials (CENTRAL), Scopus, and Web of Science databases. The reference lists of systematic reviews were screened for additional resources, and the search strategy included the following terms:

MEDLINE via PubMed: ("renal insufficiency, chronic"[MeSH Terms] OR ("renal"[All Fields] AND "insufficiency"[All Fields] AND "chronic"[All Fields]) OR "chronic renal insufficiency"[All Fields] OR ("chronic"[All Fields] AND "kidney"[All Fields] AND "disease"[All Fields]) OR "chronic kidney disease"[All Fields]) AND ("periodontal"[All Fields] OR "periodontally"[All Fields] OR "periodontically"[All Fields] OR "periodontics"[MeSH Terms] OR "periodontics"[All Fields] OR "periodontic"[All Fields] OR "periodontitis"[MeSH Terms] OR "periodontitis"[All Fields]). KK, ARR, and MDRE were the authors responsible for selecting and reading the abstracts and full articles.

### Selection criteria

English-language articles were exported into a citation program, and duplicates were removed automatically. All articles were screened by title and abstract for relevance. KK, ARR, and MDRE were the authors responsible for selecting and reading the abstracts and complete manuscripts. Full texts were then retrieved for a final decision. Case–control, cohort, and interventional studies published in English up until the search date (31 August 2024) were retrieved for screening and selection. Studies that assessed the following issues were included: (i) the association between the prevalence of CKD and periodontitis; (ii) the effect of periodontal therapy on kidney function parameters; (iii) the influence of periodontitis on all-cause mortality in CKD patients; (iv) kidney function parameters (estimated glomerular filtration rate, serum creatinine, and serum albumin) and periodontal parameters (clinical attachment level, probing pocket depth, bleeding on probing); and (v) gene polymorphisms and inflammatory parameters (cytokines, matrix metalloproteinases).

Interventional studies were assessed according to the following PICO framework: patients (P): CKD patients, intervention (I): periodontal therapy, comparison (C): comparison with a control group without CKD receiving periodontal therapy, and outcome (O) of kidney function parameters and inflammatory markers in serum. Animal studies, investigations in non-adult participants, and studies lacking a renal or periodontal parameter as an outcome variable were excluded.

### Data extraction and data presentation

Full texts of the studies that matched the inclusion criteria were screened, and the number of participants, study type, publication year, and results were extracted. All studies included were screened for the outcome variables of periodontitis diagnosis (according to either the AAP/EFP classification [36], the CDC/AAP classification [37], or the World Health Organisation (WHO) community periodontal index (CPI) [38]) and of chronic kidney disease diagnosis, which includes all stages of CKD (stages 1–5), and/or kidney failure. The Kidney Disease: Improving Global Outcomes (KDIGO) criteria were used to screen for CKD and kidney failure (KF) diagnoses [39].

## Results

**Fig. 1:**
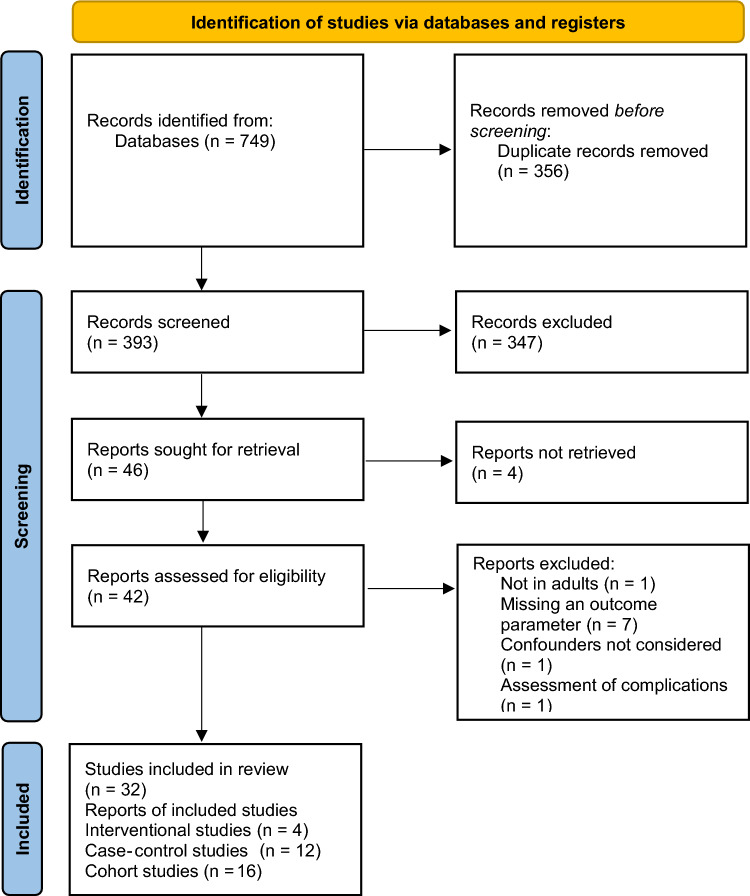
PRISMA^40^ flowchart of the identification, screening, and selection of the included studies

A total of 749 articles were identified following searches in the four online databases mentioned above. After removing the duplicates, 393 articles were screened further. The selection process of the articles is presented in Fig. 1 [40]. A total of 347 articles were excluded upon first screening by title and abstract for relevance. Eligibility of the remaining 46 articles was evaluated after full-text retrievals, and eventually 32 studies were included in the synthesis.

Four of these 32 studies (two controlled and two uncontrolled) with n = 11 up to n = 77 participants over a study period of 3–12 months reported a positive effect of non-surgical periodontal subgingival instrumentation on kidney function parameters (eGFR, serum creatinine, and albumin), vascular endothelial markers (asymmetrical dimethylarginine [ADMA]) but not on systemic inflammatory markers (Interleukin-1ß [IL-1ß], interleukin-6 [IL-6], and C-reactive protein [CRP]) (Table 1) [41–44].

**Table 1:** Tabular descriptive presentation of included interventional studies (n = 4)

Study group	Number of participants	Methods	Summary of results
Chung et al. 2022 [41]	11 haemodialysis patients	Interventional pilot study to measure the effect of periodontal therapy on kidney parameters and systemic inflammation (IL-1ß) 6 participants in test and 5 participants in control group 3 months follow-up period	No effect of periodontal therapy on systemic IL-1ß levels but only local reduction of IL-1ß levels in periodontal environment
Grubbs et al. 2020 [42]	28 participants in test (immediate) and 13 participants in control group (late) completed the study. Participants had CKD stages 1 to 3	Interventional randomised-controlled pilot study with 2:1 allocation ratio of treatment and control groups Effect of immediate or late non-surgical subgingival instrumentation on kidney and inflammatory biomarker levels were investigated and assessed at baseline, 4, 8 and 12 months	Periodontal parameters such as BOP and PPD improved more in the immediate group Vascular endothelial markers and systemic inflammation markers were reduced in both groups
Almeida et al. 2017 [43]	26 CKD stages 3 and 4 patients with periodontitis	Interventional non-controlled clinical study No control group Comparison of CKD parameters after periodontal therapy at 90 and 180 days	Increased eGFR values at 90 and 180 days Reduced ADMA levels at 180 days
Yazdi et al. 2013 [44]	77 haemodialysis patients	Interventional study to measure the influence of periodontal therapy on CRP levels in serum Comparison of serum CRP levels at baseline and 8 weeks after periodontal therapy	Post-periodontal therapy the CRP levels reduced significantly independent of the severity of periodontitis

*ADMA* asymmetrical dimethylarginine, *BOP* bleeding on probing, *CKD* chronic kidney disease, *CRP* C-reactive protein, *eGFR* estimated glomerular filtration rate, *IL-1ß* interleukin-1ß, *PPD* probing pocket depth

Twelve of the 32 studies were case–control studies that compared periodontitis prevalence and its severity, serum urea, serum creatinine, eGFR, genetic polymorphisms (monocyte chemoattractant protein-1 gene, interleukin-1, and interleukin-4), oral microbial diversity, and serum ions (calcium, potassium, phosphorus, and bicarbonate) between participants with and without CKD of different stages, including patients on kidney replacement therapy (Table 2) [45–56]. There was a wide consensus among the studies that the prevalence of periodontitis was significantly higher in CKD than in non-CKD participants, and that the severity of periodontitis influenced the kidney function parameters and serum kidney and inflammatory biomarkers. Moreover, periodontitis presented in a more severe form in patients with kidney failure and in patients undergoing kidney replacement therapies such as haemodialysis [45], and oral hygiene habits were worse in patients with elevated serum levels of urea and creatinine [46]. Salivary flow reduction and changes in saliva content in CKD patients were also reported [50]. Some studies suggest an association between genetic polymorphisms of IL-1 [53] and IL-4 [48] coding genes and MMP-1 gene promoter [55] and periodontitis in CKD patients. Increased oral microbial diversity [51] and persistence of pathological periodontal species after periodontal therapy [52] were also observed more frequently in CKD patients.

**Table 2:** Tabular descriptive presentation of included case–control studies (n = 12)

Study group	Number of participants	Methods	Summary of results
Dembowska et al. 2022. [45]	100 Haemodialysis patients and 100 healthy controls	Comparison of prevalence of periodontitis and its severity among groups	Haemodialysis group had significantly higher prevalence compared to healthy controls
Munagala et al. 2022 [46]	75 CKD patients and 75 healthy controls	Comparison of prevalence of periodontitis and serum urea, creatinine, and random glucose between groups	Significantly higher prevalence of periodontitis, poorer oral hygiene and increased serum markers were observed among CKD patients compared to controls
Ksiazek et al. 2020 [47]	150 KF patients with periodontitis, 100 KF patients without periodontitis, 190 healthy controls	Comparison of Monocyte Chemoattractant Protein (MCP)-1 -2518 (A/G) single-nucleotide polymorphism between groups	Mean MCP-1 serum levels were higher in KF patients with periodontitis compared to without periodontitis and controls
Ksiazek et al. 2019 [48]	180 KF patients with periodontitis, 82 KF patients without periodontitis, 180 healthy controls	Comparison of Interleukin-4 gene polymorphisms between groups	VNTR polymorphism in IL-4 gene was associated with periodontitis in chronic kidney disease patients
Limeres et al. 2016 [49]	40 patients on haemodialysis vs. 40 participants with eGFR above 90 ml/min	Comparison of tooth loss and periodontal parameters between groups	Haemodialysis patients presented significantly higher number of missing teeth and deeper periodontal pockets compared to control group
Anuradha et al. 2015 [50]	50 CKD patients (with or without periodontitis) vs. 50 participants without periodontitis and systemic diseases	Comparison of potassium, sodium, calcium, phosphorus, urea, and bicarbonate between groups	Reduced salivary flow and increased salivary sodium, potassium, calcium, and urea levels in CKD patients Positive correlation between the parameters and CKD disease severity
Araujo et al. 2015 [51]	14 periodontitis patients with KF vs. 13 periodontitis patients without CKD or KF	There are similar demographic and periodontal clinical parameters between groups Comparison of oral microbial diversity between groups	Diversity of periodontal microbial communities were reduced in patients with KF
Artese et al. 2012 [52]	16 stage 1–4 CKD patients with periodontitis and 14 periodontitis patients without CKD	Comparison of oral microbial species between groups at baseline and at 3 months after periodontal therapy	Higher levels of pathogenic species remained in the subgingival microbiota of CKD patients after periodontal therapy
Braosi et al. 2012 [53]	246 participants with and without CKD and periodontitis	4 groups (CKD patients with and without periodontitis, only periodontitis patients and healthy participants) Comparison of IL-1 gene polymorphisms between groups for susceptibility to CKD and periodontitis	IL1RN (*)2 allele was associated with 3 times higher risk of periodontitis in CKD patients Allele T for polymorphism IL1B + 3954 was associated with CKD in periodontitis patients
Brito et al. 2012 [54]	131 CKD stage 4–5 patients and 67 healthy individuals	Comparison of periodontal parameters among CKD stage 4 and 5 patients in pre-dialysis, continuous ambulatory peritoneal dialysis or haemodialysis and healthy participants	CKD stage 4 and 5 patients in pre-dialysis condition or undergoing haemodialysis had worse periodontal parameters compared to otherwise healthy individuals or CKD patients undergoing continuous ambulatory peritoneal dialysis
Luczyszyn et al. 2012 [55]	254 participants divided into 4 groups (with/without periodontitis and/or KF)	Comparison of matrix metalloproteinase 1–1607 (1G/2G) polymorphism between groups	There was no association between the MMP1-1607 polymorphism and periodontitis or KF
Parkar et al. 2012 [56]	152 participants undergoing haemodialysis and 152 healthy participants	Comparison of oral hygiene and periodontal parameters between haemodialysis and healthy groups	Haemodialysis group had a high severity of periodontitis and worse oral hygiene than control group

*CKD* chronic kidney disease, *CRP* C-reactive protein, *eGFR* estimated glomerular filtration rate, *HD* haemodialysis, *IL-1ß* interleukin-1ß, *KF* kidney failure *IL1RN* Interleukin-1 receptor antagonist gene

Of the 32 studies, six were cohort studies that investigated the prevalence of periodontitis and CKD within a cohort (Table 3) [57–59, 62, 64, 72]; five were prospective studies that investigated the incidence and risk of developing CKD in periodontitis and non-periodontitis patients [63, 66–69]; two reported correlations between periodontal disease and kidney function parameters between different stages of disease (n = 2) [64, 65], and finally, four analysed the association between periodontitis and kidney disease parameters using regression models (Table 3) [60, 61, 70, 71]. Investigating both the prevalence of periodontitis and its correlation with CKD, Cholewa et al. found its prevalence to be significantly higher in participants with CKD, especially in those in stages 4 and 5, and that the diagnosis of periodontal disease increased the risk of CKD [64]. Periodontal disease parameters were negatively correlated with kidney function parameter levels, and periodontitis and CKD were associated in a two-way relationship [45, 54, 55, 67, 72].

**Table 3:** Tabular descriptive presentation of included cohort studies (n = 16)

Study group	Number of participants	Methods	Summary of results
Palmeira et al. 2023 [57]	188 CKD patients	Comparison of periodontitis prevalence among CKD patients (Stages 1–3 vs. 4–5)	Periodontitis prevalence was higher in the CKD patients of Stages 4 and 5 (OR = 6.26; CI 95% = 3.13–12.52; p < 0.01)
Abou-Bakr et al. 2022 [58]	263 KF patients	Evaluation of the periodontitis prevalence and severity among participants	Duration of haemodialysis was significantly associated with worse periodontal disease parameters (increased CAL and disease severity)
Dannewitz et al. 2020 [59]	270 randomly selected CKD (stage 1 to 3) patients from larger study	Periodontitis prevalence and its severity were assessed among participants	Increased prevalence of periodontitis in CKD patients compared to general population prevalence More than 60% of severe periodontitis patients among studied cohort were not aware of their condition
Oliveira et al. 2020 [60]	180 KF patients	Periodontal parameters and oral health-related quality of life was assessed among participants with regression analysis	Periodontitis was significantly associated with psychological and physical domains and physical pain and psychological disability in its severe conditions
Schütz et al. 2020 [61]	139 CKD patients (stage 3 to 5)	Association between periodontitis and different stages of chronic kidney disease	Severe periodontitis was significantly associated with poorer kidney function in CKD (stage 3 to 5) patients
Kopic et al. 2019 [62]	80 participants (40 CKD (stage 3–5) and 40 haemodialysis patients)	Comparison of periodontal status and inflammatory cytokines between groups	Haemodialysis group showed increased levels of IL-6 and poorer periodontal status
Lertpimonchai et al. 2019 [63]	2,635 participants without chronic kidney disease at baseline	Comparison of CKD incidence in periodontitis and/or diabetes patients by mediation analysis with 1,000-replication bootstrapping	Increased severity of periodontitis affected risk of developing CKD directly and indirectly (in co-diabetics)
Cholewa et al. 2018 [64]	128 haemodialysis patients 103 dentate and 25 edentulous participants)	103 dentate and 25 edentulous participants were compared for C-reactive protein (CRP), serum albumin, calcium, phosphorus, alkaline phosphatase and parathormone including periodontal parameters	Haemodialysis patients indicated a high prevalence and severity of periodontitis compared to global prevalence Serum CRP were negatively correlated with number of teeth Periodontal pocket depth was negatively correlated with serum albumin
Ausavarungnirun et al. 2016 [65]	129 CKD (stage 2 to 5) patients	Comparison of periodontitis severity in different stages of CKD (based on eGFR values)	Severity of periodontitis increased with the increasing severity of CKD
Chen et al. 2015 [66]	100,263 participants from Annual Elderly Health Examination Program in Taiwan	Mortality and renal function were assessed among participants with and without periodontitis with a follow-up period of 3.8 years	eGFR decline and all-cause mortality were significantly more pronounced in participants with periodontitis
Grubbs et al. 2015 [67]	699 participants with preserved kidney function	Assessment of CKD incidence defined as stage 1 or higher among participants with or without periodontitis over 4-year observation period Kidney function was assessed using eGFR	Participants with severe periodontitis had fourfold higher CKD incident rate compared to participants without periodontitis
Ricardo et al. 2015 [68]	10,755 participants from NHANES III	Cohort study including periodontitis and CKD (all stages) patients All-cause and cardiovascular mortality were assessed using Cox proportional hazards model with a median follow-up of 14 years considering chronic kidney disease and periodontitis	Participants with only periodontitis or CKD had 39% higher risk of all-cause mortality and 55% higher risk of cardiovascular mortality Participants with periodontitis and CKD had more than two-fold increased risk of all-cause and cardiovascular mortality
Lee et al. 2014 [69]	35,496 participants in treatment and 141,824 participants in control group from insurance claims in Taiwan	Effect of surgical periodontal therapy on risk of developing end-stage renal disease was assessed with a follow-up period of 12 years	Risk of end-stage renal disease was lower in the treatment group than control group with an adjusted hazard ratio of 0.59 (95% CI = 0.46–0.75)
Salimi et al. 2014 [70]	13,270 participants from NHANES III	Association between periodontal parameters, eGFR, albuminuria and leucocytosis	Severe periodontitis was significantly associated with albuminuria but not with eGFR Worse periodontal parameters synergistically increased leucocytosis in CKD patients
Han et al. 2013 [71]	15,729 adults from Korean National Health and Nutritional Examination Surveys IV and V	Association between periodontitis and CKD markers such as eGFR, proteinuria and haematuria were assessed	Periodontitis was significantly associated with decreased eGFR, proteinuria and haematuria in the study population
Ioannidou et al. 2013 [72]	3686 participants from NHANES III	Prevalence of periodontitis was compared between participants with and without CKD	Periodontitis was significantly more prevalent among severely and moderately reduced eGFR participants compared to mildly to not reduced eGFR participants

*CAL* clinical attachment loss, *CRP* C-reactive protein, *CKD* chronic kidney disease, *eGFR* estimated glomerular filtration rate, *KF* kidney failure, *NHANES* National Health and Nutrition Examination Survey

## Discussion

In this scoping review, the relationship between CKD and periodontitis was investigated from the perspective of genetic, microbiological and molecular factors, periodontal disease parameters, and proxies to summarise the mechanisms underlying the two-way association between the diseases. In spite of the heterogeneity among the studies, the cumulative results from this and other reviews [34, 73, 74] indicate that periodontitis is more frequent in patients with CKD. This high prevalence can be explained by the common risk hypothesis of chronic inflammatory diseases [75]. Smoking and diabetes mellitus are both risk factors for periodontitis and CKD [76, 77]; thus, the presence of a risk factor may increase the overall likelihood of co-diagnosis of an additional chronic inflammatory disease [78]. Besides, the initially existing disease may increase overall systemic inflammation, which is observed in both periodontitis and CKD, resulting in the exacerbation of the other condition [79, 80]. However, in individuals who may share similar risk factors, additional circumstances, such as oral hygiene habits, microbiome changes, and genetic polymorphisms, can play a significant role in the occurrence of these diseases [81].

Patients with CKD may present poor oral hygiene for a number of reasons, including the psychological and physical inability to maintain good oral hygiene due to their reduced quality of life) [60] or changes in salivary content and function due to impaired kidney function, such as an increase in urea concentration in saliva and salivary pH changes [50]. Interventions to address oral hygiene habits and salivary supplements should be considered when treating patients with co-morbidities [82]. It has also been suggested that oral microbiota changes are affected by salivary pH changes, resulting in an increased oral microbial diversity and the persistence of oral pathological species in CKD patients. In periodontitis, an increased microbial diversity (which can also be assessed as alpha diversity) is associated with a higher level of dysbiotic changes in the local microbiota (unlike gastrointestinal dysbiosis-related diseases) and thus an increased risk of disease [25, 83, 84]. Microbial diversity may be an underlying mechanism of an association between periodontitis and CKD. As oral hygiene habits alone cannot be the sole cause of the onset of periodontitis, and its progression is also immune-modulated, individual genetic factors and gene expression profiles also appear to play a major role in its pathophysiology of CKD [22]. This explains the variability in the prevalence and incidence among the cohorts studied, who may share similar environmental and behavioural risk factors for CKD and/or periodontitis [85, 86]. For instance, the increased incidence of CKD and aggressive periodontitis in individuals of African origin suggests that genetic predisposition might be an important contributor to the emergence of these conditions [87, 88]. In addition, the prevalence of *Il-1* and *Il-4* gene polymorphisms was higher in periodontitis patients with CKD than in those without CKD [48, 53].

The most important limitation of this study is the heterogeneity of the criteria used to classify periodontitis in the studies included in this scoping review; this may have influenced the prevalence of the condition in the groups [89].

The association between CKD and periodontitis has been demonstrated in many different geographical regions, indicating that this is a global health concern [90, 91]. The increasing global prevalence of periodontitis and CKD also raises the risk of cardiovascular diseases, since these two conditions are significantly associated with adverse cardiovascular events [26, 92]. A recent cross-sectional study showed a positive correlation between the incidence of periodontitis and CKD (OR = 2.14) [93]. The authors indicated the existence of a common risk factor mediated by immune cells, namely the expression of CD64 monocytes An interdisciplinary, personalised approach to disease screening, prevention, and management is necessary to reduce the global burden of disease, not only that of periodontitis and CKD but of cardiovascular diseases as well [90, 94].

The One Health concept was introduced in the mid-twentieth century and since then has underpinned many efforts to improve the overall health and well-being of the world’s population by addressing health from a broader and holistic perspective, considering wider determinants of health, all the organisms on earth, and the environment [95]. With a view to improving population health by addressing all contributing factors to disease initiation and progression, healthcare provision should cover all aspects of health, including oral and periodontal health. Thus, interdisciplinary work involving the disciplines of medicine and dentistry should be promoted. Interventional studies conducted in patients with type II diabetes mellitus and periodontitis provide excellent examples of how interdisciplinary work combining the fields of medicine and dentistry can boost overall health. A meta-analysis of interventional studies [96] reported that periodontal therapy may significantly improve HbA1c levels in diabetic patients, thus contributing to the overall management of their diabetic status. It has been proposed that the mechanisms underlying this positive effect of periodontal therapy in diabetic patients are the reductions in low-grade chronic systemic inflammation and bacterial load [97]. A significant association has been reported between type II diabetes mellitus and low-grade systemic inflammation, similar to the pathophysiology of CKD [98]; consequently, it is reasonable to assume that periodontal therapy could also improve the overall health of CKD patients. Investigating the effect of periodontal therapy on kidney function, Almeida et al. concluded that kidney function (evaluated as eGFR) improved after periodontal therapy, as we found in our review [43]. Therefore, an interdisciplinary approach combining the fields of nephrology and periodontology could alleviate the disease burden for individuals with CKD. A recent study conducted by the Centers for Disease Prevention and Control (CDC) in six US states concluded that oral and dental health were considered distinct from general health, and that this view represented a significant barrier to implementing programs to improve oral health in individuals with chronic diseases [98]. Another significant obstacle to the integration of oral health into primary care is the lack of political will [99, 100]. Despite the challenges, the pilot study conducted by the CDC concluded that close collaboration between medical and dental disciplines can enhance financing for screening, training, and referrals for conditions that share risk factors with chronic diseases, and thus their management as well. The results of the present study also highlight the need to conduct randomised-controlled interventional studies and large longitudinal cohort studies. Both containing healthy participants and patients with CKD or periodontitis to measure the degree of the association and risk assessment, and to test whether periodontal treatment improves a kidney function parameters and vice versa. Policymakers should consider implementing and facilitating access to oral health services for individuals diagnosed with a systemic chronic inflammatory disease or who may be at a high risk of developing one.

## Conclusions

In the field of periodontal medicine, low-grade systemic inflammation plays an important role in the development of certain chronic pathologies. Generalised active periodontitis can be considered a factor related to inflammation throughout the body and also is a contributing risk factor for developing kidney failure.

The present scoping review indicates that periodontitis is observed more frequently in CKD patients, and intervention studies have suggested that periodontal therapy may improve some kidney function parameters. Nonetheless, future longitudinal studies are needed to investigate possible cause and effect in this two-way relationship.

## Data Availability

For those interested, the datasets used and/or analysed during the present study can be obtained by contacting the first or corresponding author.
